# National Estimates of Short- and Longer-Term Hospital Readmissions After Major Surgery Among Community-Living Older Adults

**DOI:** 10.1001/jamanetworkopen.2024.0028

**Published:** 2024-02-28

**Authors:** Yi Wang, Linda Leo-Summers, Brent Vander Wyk, Kendra Davis-Plourde, Thomas M. Gill, Robert D. Becher

**Affiliations:** 1 Department of Internal Medicine, Yale School of Medicine, New Haven, Connecticut; 2 Department of Biostatistics, Yale School of Public Health, New Haven, Connecticut; 3 Department of Surgery, Yale School of Medicine, New Haven, Connecticut

## Abstract

**Importance:**

Nationally representative estimates of hospital readmissions within 30 and 180 days after major surgery, including both fee-for-service and Medicare Advantage beneficiaries, are lacking.

**Objectives:**

To provide population-based estimates of hospital readmission within 30 and 180 days after major surgery in community-living older US residents and examine whether these estimates differ according to key demographic, surgical, and geriatric characteristics.

**Design, Setting, and Participants:**

A prospective longitudinal cohort study of National Health and Aging Trends Study data (calendar years 2011-2018), linked to records from the Centers for Medicare & Medicaid Services (CMS). Data analysis was conducted from April to August 2023. Participants included community-living US residents of the contiguous US aged 65 years or older who had at least 1 major surgery from 2011 to 2018. Data analysis was conducted from April 10 to August 28, 2023.

**Main Outcomes and Measures:**

Major operations and hospital readmissions within 30 and 180 days were identified through data linkages with CMS files that included both fee-for-service and Medicare Advantage beneficiaries. Data on frailty and dementia were obtained from the annual National Health and Aging Trends Study assessments.

**Results:**

A total of 1780 major operations (representing 9 556 171 survey-weighted operations nationally) were identified from 1477 community-living participants; mean (SD) age was 79.5 (7.0) years, with 56% being female. The weighted rates of hospital readmission were 11.6% (95% CI, 9.8%-13.6%) for 30 days and 27.6% (95% CI, 24.7%-30.7%) for 180 days. The highest readmission rates within 180 days were observed among participants aged 90 years or older (36.8%; 95% CI, 28.3%-46.3%), those undergoing vascular surgery (45.8%; 95% CI, 37.7%-54.1%), and persons with frailty (36.9%; 95% CI, 30.8%-43.5%) or probable dementia (39.0%; 95% CI, 30.7%-48.1%). In age- and sex-adjusted models with death as a competing risk, the hazard ratios for hospital readmission within 180 days were 2.29 (95% CI, 1.70-3.09) for frailty and 1.58 (95% CI, 1.15-2.18) for probable dementia.

**Conclusions and Relevance:**

In this nationally representative cohort study of community-living older US residents, the likelihood of hospital readmissions within 180 days after major surgery was increased among older persons who were frail or had probable dementia, highlighting the potential value of these geriatric conditions in identifying those at increased risk.

## Key Points

**Question:**

What are nationally representative estimates of hospital readmissions within 30 and 180 days after major surgery among community-living US residents aged 65 years or older?

**Findings:**

In this population-based cohort study of 1477 individuals, rates of hospital readmission were 11.6% within 30 days and 27.6% within 180 days. Readmission rates at 180 days were 36.9% for persons who were frail and 39.0% for those with probable dementia.

**Meaning:**

The findings of this study suggest that frailty and probable dementia, 2 important geriatric conditions, have potential value in identifying increased risk of longer-term hospital readmissions after major surgery in community-living older US residents.

## Teaser

This cohort study estimates national 30- and 180-day hospital readmission rates following major surgery in older community-living individuals.

## Introduction

In the US, hospital readmissions are common, costly, and increasingly used in pay-for-performance metrics.^1,2^ There were 3.8 million 30-day, all-cause adult hospital readmissions in 2018, totaling over $50 billion in hospital readmission costs.^3^ Medicare beneficiaries aged 65 years or older account for most of these readmissions,^3^ and the Centers for Medicare & Medicaid Services (CMS) has dedicated substantial efforts to reducing readmissions and their financial toll. In 2010, the CMS Hospital Readmissions Reduction Program (HRRP) was established as part of the Affordable Care Act,^4^ imposing fiscal penalties on hospitals with higher-than-expected 30-day all-cause readmission rates. The HRRP program initially included only medical conditions^2^ but was later expanded to cover 3 types of surgical operations (coronary artery bypass graft and total hip and knee arthroplasty), with plans by the CMS to potentially expand the HRRP program to include more operations across additional surgical specialties.^5^

Understanding hospital readmissions after major surgery is, therefore, important for multiple stakeholders, including the CMS, surgeons, and clinicians caring for older adults, hospitals and hospital administrators, and federal decision-makers who develop and implement health policy. Yet, nationally representative estimates of hospital readmissions within 30 days (short-term) and 180 days (longer-term) after major surgery in older persons are lacking. This is problematic for 4 reasons. First, 30-day readmission is an important metric to evaluate hospital performance in federal programs.^6,7^ While nationally representative estimates of mortality after major geriatric surgery have recently been reported,^8^ comparable information on hospital readmission is not available. Second, major surgery often requires an extended recovery period among older persons,^9,10^ placing them at increased risk for hospital readmission beyond 30 days. Both the National Institutes of Health and the American College of Surgeons have argued that evaluating longer-term outcomes following surgery is critical to fully comprehend surgical quality and safety.^11^ Nonetheless, relatively little is known about rehospitalizations after major surgery beyond 30 days.^2,12,13^ Third, geriatric-specific conditions, such as frailty and dementia, are known to impact postoperative mortality, yet estimates of hospital readmission by geriatric phenotypes are not well defined.^14^ Such information would provide additional clarity for clinicians, patients, and families to better understand the recovery process after major surgery. Fourth, prior estimates of hospital readmissions after major geriatric surgery in the US provide an incomplete picture because they did not include the growing Medicare Advantage (MA) population, relying instead solely on beneficiaries with fee-for-service (FFS) Medicare.^15^

To address these gaps in knowledge, the current study had 2 main objectives: first, to calculate nationally representative rates of hospital readmissions within 30 and 180 days after major surgery among community-living older US residents; and second, to examine whether these estimates differ according to key demographic, surgical, and geriatric characteristics, including frailty and dementia. To achieve these objectives, we used data from the National Health and Aging Trends Study (NHATS), linked to records from the CMS. These records included hospitalizations from participants with both FFS and MA, providing a comprehensive and novel data set for our analysis.

## Methods

### Study Design and Data Sources

NHATS is a prospective nationally representative longitudinal cohort study of Medicare beneficiaries in the contiguous US (excluding Alaska, Hawaii, and Puerto Rico). A complete description of the cohort has been provided elsewhere.^16,17^ Briefly, NHATS uses the Medicare enrollment file as the sampling frame, and the 2011 cohort represents Medicare enrollees aged 65 years or older as of September 30, 2010. The baseline survey, which was completed from May through November 2011, yielded a sample of 8245 persons with a 71.3% weighted response rate. Follow-up assessments have been completed annually by trained research staff. These assessments include extensive demographic, socioeconomic, and high-quality patient-centered phenotypic data that are not available in administrative data sets. The NHATS data were linked to CMS records (FFS and MA) to identify participants who underwent major surgery. NHATS was approved by The Johns Hopkins University Institutional Review Board, and all participants provided informed consent. The current study, which was approved by the Yale University Institutional Review Board, followed the Strengthening the Reporting of Observational Studies in Epidemiology (STROBE) reporting guideline. The study was conducted from calendar years 2011 to 2018.

### Study Population

The study population included NHATS participants who subsequently had at least 1 major surgery, defined as any procedure in an operating room requiring the use of general anesthesia for a nonpercutaneous, nonendoscopic invasive operation. This definition, which has been previously operationalized and implemented,^8,18,19,20^ is consistent with other definitions of high-risk surgery in older persons.^21,22^ We categorized each operation into 1 of 6 types: (1) musculoskeletal, (2) abdominal (including gastrointestinal), (3) vascular (including endovascular, non–coronary bypass grafts, and amputations), (4) neurologic (including brain and spine), (5) cardiothoracic, and (6) other (including major endocrine, gynecologic, urologic, breast, plastic, otolaryngologic, and transplant surgery). Major operations were also classified as elective or nonelective based on a CMS indicator variable.

### Assembly of Analytic Sample

Major operations were included through December 2018. Participants could contribute more than 1 observation to the analysis based on the following criteria: (1) participant had to be community living at the time of the prior annual assessment, (2) participant was not admitted from a nursing home, (3) observation represented the first major surgery within the annual interval, (4) participant did not contribute a major surgery within the prior 3 months because the prior observation may have altered key participant characteristics from the prior annual assessment, and (5) participant had to be discharged from the hospital without hospice services.

eFigure 1 in Supplement 1 shows the assembly of the analytic sample. Of the 3015 major operations, 1235 were excluded, leaving 1780 observations from 1477 participants in the analytic sample. To avoid overlapping follow-up intervals, 19 additional observations were excluded for the 180-day readmission analysis.

### Participant Characteristics

At baseline, information was collected on demographic characteristics, including age, sex, race and ethnicity, and education; 9 chronic conditions (listed in the Table), and 2 geriatric conditions. Race and ethnicity were included to allow for the evaluation of racial and ethnic disparities and health inequities. Race and ethnicity were self-reported and included as part of the sociodemographic description of the cohort. Race data were collected in the following categories: Alaska Native, American Indian, Black or African American, Native Hawaiian, Pacific Islander, White, and Other. Ethnicity data were collected in the following categories: Cuban American, Mexican American or Chicano, Puerto Rican, and other. The Other category includes participants who reported their race and ethnicity as American Indian, Asian, Native Hawaiian, Pacific Islander, other, do not know, or more than 1 race and ethnicity. The geriatric conditions were classified as nonfrail, prefrail, and frail using the Fried phenotype assessment^23^ and as no dementia, possible dementia, and probable dementia according to a validated assessment strategy.^24,25^ Data on frailty and dementia were updated during the annual assessments and were 100% complete. Medicare type and Medicaid eligibility were obtained from the CMS records.

**Table.** Characteristics of Major Operations Contributed by Community-Living Participants From 2011 to 2018^a^

Characteristic	No. (%)		
	All operations	Elective surgery	Nonelective surgery
No. of observations	1780	993	787
Weighted No. of observations^b^	9 556 171	5 697 291	3 858 880
Age, mean (SD), y	79.5 (7.0)	78.0 (6.4)	81.4 (7.4)
Age group, y			
65-69	117 (6.6)	84 (8.5)	33 (4.2)
70-74	380 (21.3)	244 (24.6)	136 (17.3)
75-79	444 (24.9)	278 (28.0)	166 (21.1)
80-84	395 (22.2)	218 (22.0)	177 (22.5)
85-89	289 (16.2)	127 (12.8)	162 (20.6)
≥90	155 (8.7)	42 (4.2)	113 (14.4)
Sex			
Male	786 (44.2)	436 (43.9)	350 (44.5)
Female	994 (55.8)	557 (56.1)	437 (55.5)
Race and ethnicity^c^			
Non-Hispanic Black	312 (17.5)	148 (14.9)	164 (20.8)
Hispanic	68 (3.8)	36 (3.6)	32 (4.1)
Non-Hispanic White	1336 (75.1)	780 (78.5)	556 (70.6)
Other	64 (3.6)	29 (2.9)	35 (4.4)
Education			
Less than high school	395 (22.4)	191 (19.4)	204 (26.2)
High school or equivalent	484 (27.4)	269 (27.3)	215 (27.6)
Beyond high school	888 (50.3)	527 (53.4)	361 (46.3)
Medicare type			
Medicare fee-for-service	1228 (69.0)	695 (70.0)	533 (67.7)
Medicare Advantage	552 (31.0)	298 (30.0)	254 (32.3)
Medicaid eligible	276 (15.5)	124 (12.5)	152 (19.3)
No. chronic conditions, mean (SD)^d^	2.8 (1.4)	2.8 (1.4)	2.8 (1.4)
Frailty phenotype			
Nonfrail	441 (24.8)	284 (28.6)	157 (19.9)
Prefrail	906 (50.9)	504 (50.8)	402 (51.1)
Frail	433 (24.3)	205 (20.6)	228 (29.0)
Dementia status			
No dementia	1397 (78.5)	843 (84.9)	554 (70.4)
Possible dementia	188 (10.6)	88 (8.9)	100 (12.7)
Probable dementia	195 (11.0)	62 (6.2)	133 (16.9)
Type of surgery			
Musculoskeletal	777 (43.7)	424 (42.7)	353 (44.9)
Abdominal	305 (17.1)	112 (11.3)	193 (24.5)
Vascular	196 (11.0)	119 (12.0)	77 (9.8)
Cardiothoracic	169 (9.5)	107 (10.8)	62 (7.9)
Neurologic	153 (8.6)	109 (11.0)	44 (5.6)
Other^e^	180 (10.1)	122 (12.3)	58 (7.4)

^a^ Unless otherwise stated, the data are presented as unweighted values. The values for number of chronic conditions, frailty phenotype, and dementia status were obtained during the annual assessment immediately before the operation. Some percentages may not sum to 100 because of missing data. The 1780 observations were contributed by 1477 persons, as described in the Methods Section.

^b^ Estimates after applying National Health and Aging Trends Study analytic sampling weights to the total count of hospital admissions for major surgery.

^c^ Race and ethnicity were self-reported and included as part of the sociodemographic description of the cohort. Race data were collected in the following categories: Alaska Native, American Indian, Black or African American, Native Hawaiian, Pacific Islander, White, and Other. Ethnicity data were collected in the following categories: Cuban American, Mexican American or Chicano, Puerto Rican, and other. Race and ethnicity data were combined as shown. The Other category includes participants who reported their race and ethnicity as American Indian, Asian, Native Hawaiian, Pacific Islander, other, do not know, or more than 1 race and ethnicity.

^d^ Includes 9 self-reported, physician-diagnosed chronic conditions, including heart attack, high blood pressure, arthritis, osteoporosis, diabetes, lung disease, stroke, cancer, and hip fracture since age 50 years.

^e^ Includes major endocrine, gynecologic, urologic, breast, plastic, otolaryngologic, and transplant surgery.

### Hospital Readmissions

The first hospital readmission up to 180 days after major surgery was ascertained from the linked CMS records. Information was obtained on principal diagnosis, whether the readmission was unplanned (admitted through emergency department) or for major surgery, and length of hospital stay. The principal diagnoses for these readmissions were categorized within clinically meaningful groups by aggregating individual *International Classification of Disease* (*ICD*) codes though the use of the Clinical Classifications Software system.^26,27,28^

### Statistical Analysis

Descriptive characteristics were assessed for all major operations and for elective and nonelective procedures. For each person-year of NHATS data, we used the specific sampling weights that adjust for differential probabilities of selection and nonresponse.

NHATS-weighted all-cause hospitalization readmission rates within 30 and 180 days were generated according to key demographic, surgical, and geriatric characteristics. Weighted Kaplan-Meier curves were used to determine the cumulative hazard of hospital readmissions over 180 days, stratified by key demographic, surgical, and geriatric characteristics. Adjusted risks for hospital readmissions were evaluated at both 30 and 180 days. The models for age were adjusted for sex, the models for sex were adjusted for age, and all other models were adjusted for age and sex. As per convention, 30-day readmission was evaluated as a dichotomous outcome, and risk ratios were generated through the log link function.^29^ Given the extended time frame, readmission within 180 days was evaluated as a time-to-event outcome. To account for the competing risk of death, Fine-Gray models using subdistribution hazards regressions were used, yielding subdistribution hazard ratios.^30^ To enhance clinical interpretability, we computed restricted mean readmission times for statistically significant subgroups and their corresponding reference group from the adjusted competing hazard models. The differences between these values can be interpreted as differences in time to hospital readmission. For ease of presentation, we use the term risk when describing the risk ratio and hazard ratio results. All analyses were performed with Stata, version 17.0 (StataCorp LLC) and SAS, version 9.4 (SAS Institute Inc). Figures were created and finalized in R statistical software, version 4.2.2 (R Foundation for Statistical Computing). Data were analyzed from April 10 to August 28, 2023. A 2-sided, unpaired *P* < .05 value was considered statistically significant.

## Results

The Table provides the characteristics of the analytic sample. Overall, 1780 major operations, representing 9 556 171 survey-weighted observations, were included. The mean (SD) age was 79.5 years (7.0 years), with 56% being female and 44% male. A total of 1336 patients (75.1%) were non-Hispanic White, 879 (49.8%) had a high school education or less, 1228 (69.0%) had fee-for-service Medicare coverage, and 276 (15.5%) were eligible for Medicaid. The 2 most common types of surgery were musculoskeletal and abdominal. Relative to those who had elective surgery, participants who underwent nonelective surgery were older, had lower educational attainment, and were more likely to be frail and cognitively impaired.

eTable 1 in Supplement 1 provides the weighted rates of hospital readmission after major surgery according to relevant demographic, surgical and geriatric subgroups. The readmission rates were 11.6% (95% CI, 9.8%-13.6%) at 30 days and 27.6% (95% CI, 24.7%-30.7%) at 180 days. Over the 180-day follow-up period, there were 52 deaths without readmissions (2.3%; 95% CI, 1.6%-3.2%), and the median time to readmission was 44 (IQR, 15-105) days. The highest readmission rates within 180 days were observed among participants aged 90 years or older (36.8%; 95% CI, 28.3%-46.3%), those undergoing vascular surgery (45.8% 95% CI, 37.7%-54.1%), and persons with frailty (36.9%; 95% CI, 30.8%-43.5%) or probable dementia (39.0%; 95% CI, 30.7%-48.1%). Additional information, including rates of hospital readmissions that were unplanned or for major surgery, is provided in eTable 2 in Supplement 1. As shown in eFigure 2 in Supplement 1, septicemia, device complications, procedural complications, and congestive heart failure were the 4 most common diagnoses for hospital readmission within both 30 and 180 days.

**Figure 1.**
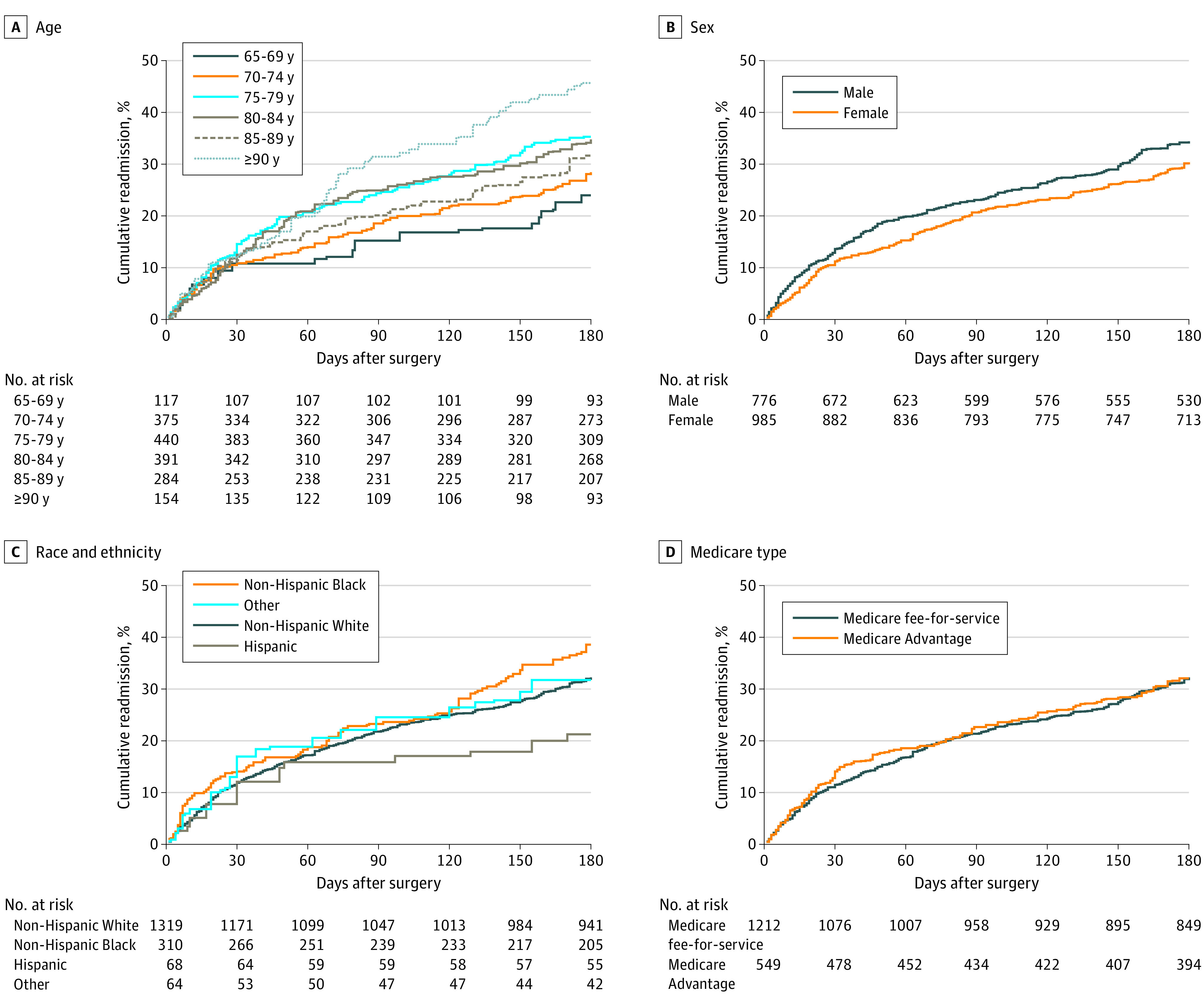
Cumulative Hazard of Hospital Readmissions During 180 Days After Major Surgery by Demographic Characteristics National Health and Aging Trends Study–weighted Kaplan-Meier curves are shown. Numbers at risk represent unweighted values.

**Figure 2.**
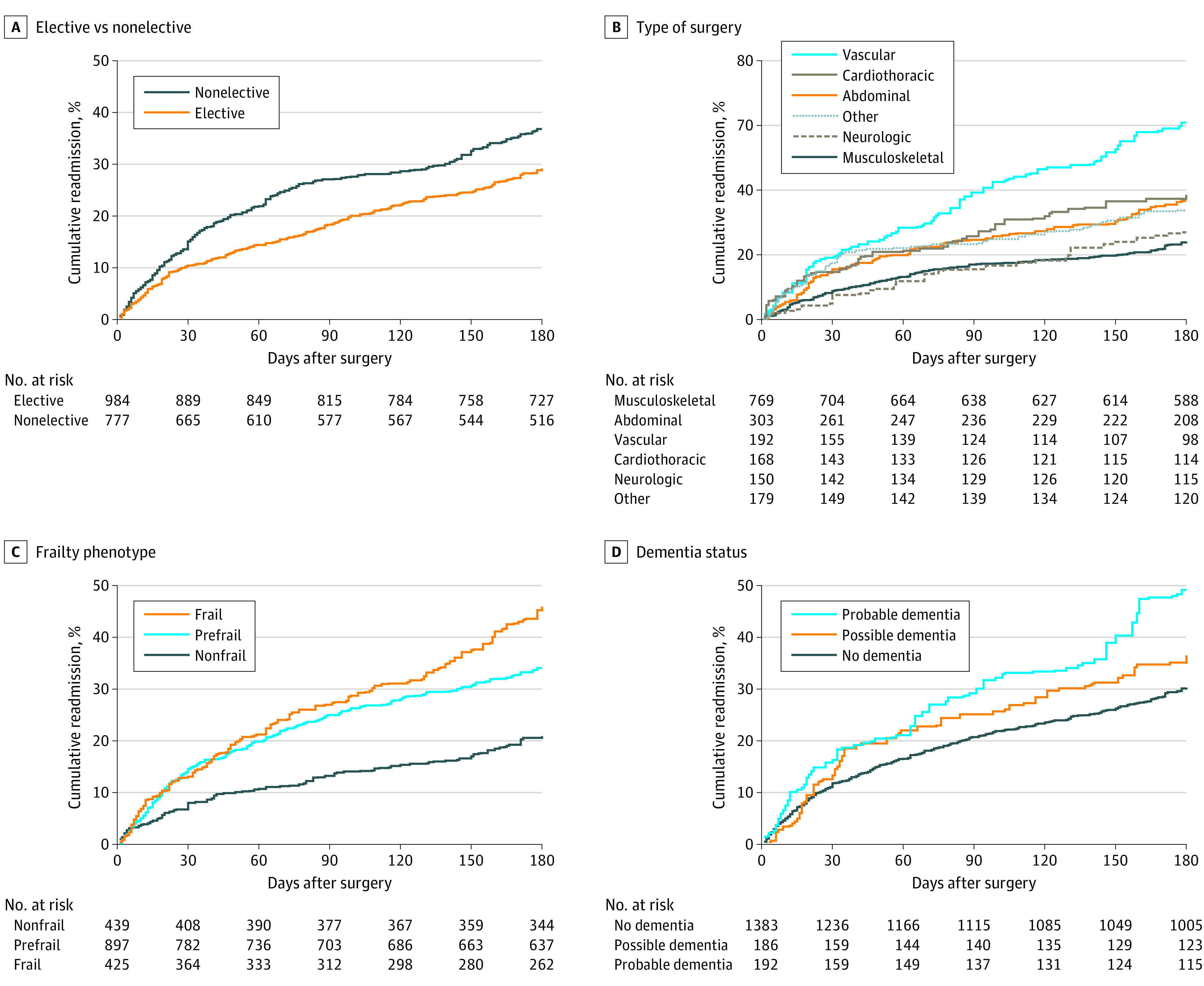
Cumulative Hazard of Hospital Readmissions During 180 Days After Major Surgery by Surgical and Geriatric Characteristics National Health and Aging Trends Study–weighted Kaplan-Meier curves are shown. Numbers at risk represent unweighted values.

Figure 1 shows the cumulative hazard of hospital readmissions during 180 days after major surgery by demographic characteristics. Readmissions were highest for participants aged 90 years or older and lowest for Hispanic individuals, with little difference by sex or Medicare Type. The corresponding results for the surgical and geriatric characteristics are provided in Figure 2. The cumulative hazard of hospital readmissions was higher for nonelective than elective surgery, was highest for vascular and lowest for musculoskeletal procedures, and showed the highest values for frailty and probable dementia and lowest values for nonfrailty and no dementia.

**Figure 3.**
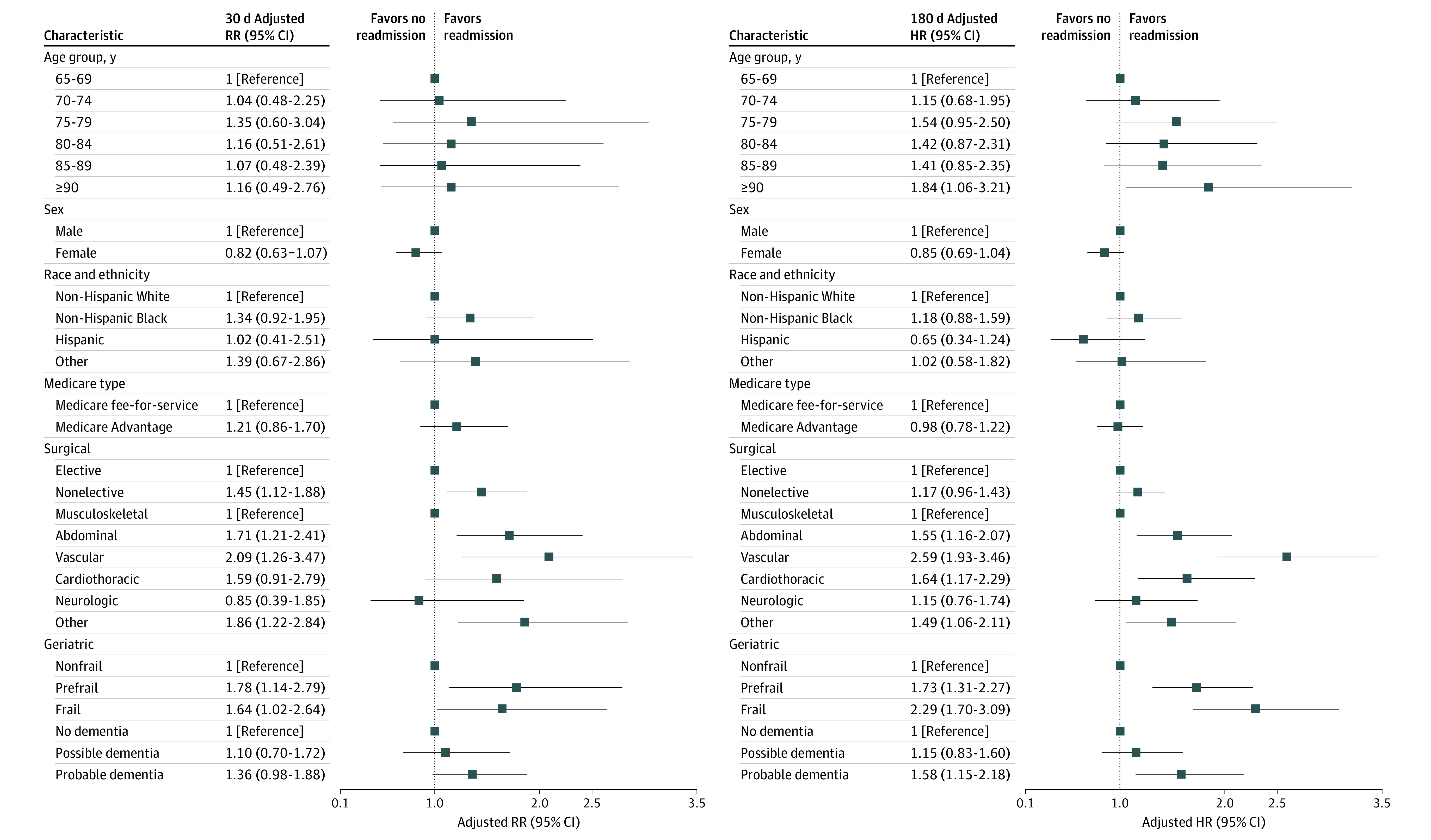
Adjusted Risks for Hospital Readmissions After Major Surgery According to Demographic, Surgical, and Geriatric Characteristics The models for age were adjusted for sex, whereas the models for sex were adjusted for age. All other models were adjusted for age and sex. Adjusted hazard ratios (HRs) were obtained from Fine-Gray models, accounting for the competing risk of death and sampling weights. RR indicates risk ratio.

Figure 3 provides the adjusted risks for hospital readmissions, according to the demographic, surgical, and geriatric characteristics. Compared with participants aged 65 to 69 years, those aged 90 years or older had an 84% increased adjusted risk for readmissions within 180 days. Otherwise, no statistically significant differences were observed within either 30 or 180 days for the demographic characteristics, including sex, race and ethnicity, and Medicare type. Hospital readmissions were significantly more likely at 30 days, but not within 180 days, for nonelective than elective operations. Relative to musculoskeletal operations, the risk for readmissions was significantly increased at both 30 and 180 days for abdominal, vascular, and other operations and within 180 days alone for cardiothoracic surgery. Among these surgical categories, vascular surgery consistently demonstrated the highest adjusted risks at both time points. For the geriatric conditions, the risk for readmissions was significantly increased at both 30 and 180 days for participants who were prefrail or frail compared with those who were nonfrail, but was increased within 180 days only for participants who had probable dementia vs no dementia. The age- and sex-adjusted hazard ratios for 180-day hospital readmissions were 2.29 (95% CI, 1.70-3.09) for frailty and 1.58 (95% CI, 1.15-2.18) for probable dementia.

**Figure 4.**
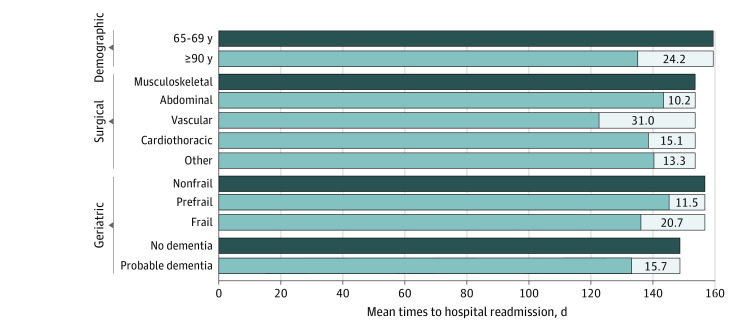
Restricted Mean Times to Hospital Readmission Within 180 Days for Relevant Demographic, Surgical, and Geriatric Subgroups Values are provided for each of the statistically significant subgroups and respective reference group (dark blue bar) from the adjusted Fine-Gray models, along with the corresponding differences in mean readmitted time..

The restricted mean times to hospital readmission within 180 days for the statistically significant subgroups and respective reference group from the adjusted hazard models are provided in Figure 4. The largest differences compared with the reference group were observed for age 90 years or older (24.2 days), vascular surgery (31.0 days), and frailty (20.7 days).

## Discussion

In this nationally representative sample of community-living older US residents, we estimated the occurrence of hospital readmissions within 30 days (short-term) and 180 days (longer-term) after major surgery and evaluated whether these population-based estimates differ on the basis of key demographic, surgical, and geriatric characteristics. We found that nearly 1 of every 8 community-living older persons had a hospital readmission within 30 days after major surgery, representing approximately 1.1 million older individuals, and more than 1 of 4 had a readmission within 180 days, representing approximately 2.6 million older individuals. The highest readmission rates within 180 days were observed among persons with frailty or probable dementia, participants aged 90 years or older, and those undergoing vascular surgery. Taken together, our findings suggest that the occurrence of hospital readmissions within 180 days after major surgery varies substantially across distinct subgroups of older persons and underscores the potential value of geriatric conditions such as frailty and dementia in identifying increased risk.

Older persons undergoing major surgery represent a large and growing population^18,31^ at increased risk of postoperative complications and hospital readmission.^32,33^ Despite this, currently available estimates of hospital readmissions after major surgery in older persons are inadequate for several reasons. Prior studies have been limited to a subset of specific surgical procedures^2,9,34,35,36^ or age groups,^9,37,38,39^ are often restricted to a single institution^40,41^ or are not otherwise nationally representative,^39,42,43^ lack data beyond 30 days,^2,12,39,44^ or have not evaluated clinically relevant geriatric subgroups.^10,35,43,45^ Furthermore, prior estimates of hospital readmission based on CMS data^2,15,46^ were based solely on FFS beneficiaries. By linking data from a well-phenotyped and nationally representative cohort of community-living older US residents to CMS records, including both FFS and MA beneficiaries, we were able to address these deficiencies and, in turn, generate a robust set of population-based estimates of hospital readmission after major surgery.

Hospital readmission is an important quality and safety metric that is used by the CMS to evaluate hospital performance.^6,7^ The HRRP,^7^ for example, is a value-based purchasing program^47^ that imposes financial penalties on hospitals with higher-than-expected readmissions within 30 days of discharge for a core group of common conditions and operations. Despite this focus on short-term readmissions, evidence has shown that most older persons require a longer recovery period after major surgery^19^ and that the risk of hospital readmission often extends to at least 6 months.^9,10,35^

The current study, which provides estimates of both 30- and 180-day hospital readmissions, adds to a growing body of research highlighting the importance of both geriatric-specific phenotypes and longer-term outcomes among older persons undergoing major surgery.^8,18,21,48^ We found substantial variations in rates and hazards of hospital readmissions within 180 days by both frailty and dementia. Readmission rates were 36.9% for frail participants (compared with 18.9% for nonfrail) and 39.0% for those with probable dementia (compared with 26.1% for no dementia), corresponding to increases in hazards that were 2.29- and 1.58-fold higher in these vulnerable subgroups of older persons. On an absolute basis, the mean times to hospital readmission during the 180-day follow-up period were 20.7 and 16 days shorter for participants with frailty and probable dementia, respectively, compared with their less vulnerable counterparts.

These findings reenforce the importance of enhanced preoperative recognition of frailty and dementia in older adults^49^ and may inform patient and family expectations—and thus surgical decision making—about postoperative trajectories in the setting of these geriatric conditions. Our findings also align well with recommendations of the Geriatric Surgery Verification Program of the American College of Surgeons,^50^ which stress the importance of frailty and cognitive impairment to geriatric surgery outcomes.

In addition to geriatric conditions, our findings provide important new information about the postoperative hospital readmission experience of older US residents across multiple demographic and surgical groups. Participants aged 90 years or older had the highest 180-day readmission rate (36.8%) across all age groups, corresponding to an adjusted increase in hazard of 84% compared with those aged 65 to 69 years. Patients aged 90 years or older represent a highly vulnerable surgical subgroup, with more comorbidities and higher rates of postoperative complications than other geriatric age groups,^49,51,52,53^ which partially explains their much higher hazard of hospital readmissions within 180 days. We also found that participants undergoing vascular surgery had the highest rates of both short- and longer-term hospital readmissions, with nearly 1 of 2 individuals being readmitted within 180 days. These findings add a longer-term context to the literature on vascular surgery, which is known to involve complex procedures with a significantly increased risk of short-term postoperative complications and more planned 30-day readmissions compared with other surgical disciplines.^54,55,56^ In addition, we found that nonelective surgery conferred a significantly increased risk of 30-day but not 180-day hospital readmission. These findings support that older patients undergoing nonelective surgery are a unique population compared with their counterparts undergoing elective surgery in the short term,^18,45^ but suggest that their subsequent relative vulnerability does not increase over time.

### Strengths and Limitations

Three unique features enhance the generalizability, validity, and applicability of our findings. First, by integrating data from NHATS and CMS (both FFS and MA), we were able to produce nationally representative estimates of hospital readmissions after major surgery among older US residents in the contiguous US states. Second, we used a well-established definition of major surgery in older persons, one that is clearly defined, clinically relevant, widely accepted, and encompasses a wide array of surgical disciplines.^8,18,21,48^ Third, we provide the estimates of hospital readmissions within both 30 and 180 days among clinically relevant geriatric subgroups defined by frailty and dementia.

Our findings should be interpreted in the context of potential limitations. First, information was not available on the reasons for surgery and postoperative complications—2 factors that likely contribute to hospital readmissions. Although beyond the scope of the current study, data on complications could inform the association between major surgery and hospital readmissions.^57^ Second, major operations beyond 2018 could not be evaluated because CMS data linked to NHATS were not yet available. To our knowledge, the standards of hospital performance in federal programs and postoperative care have not changed appreciably during the past 4 to 5 years. While the advent of the American College of Surgeons’ Geriatric Surgery Verification Quality Improvement Program in 2019 has brought greater national attention to geriatric surgery, only a small number of hospitals participate in the program.^58^ Third, because NHATS included participants from only the 48 contiguous US states, we cannot comment about the rates of hospital readmissions after major surgery among older persons from Alaska, Hawaii, or Puerto Rico.

## Conclusions

In this nationally representative, longitudinal cohort study, nearly 1 of 8 community-living older US residents had a hospital readmission within 30 days after major surgery, and more than 1 of 4 had a readmission within 180 days. The likelihood of hospital readmissions within 180 days after major surgery was increased among older persons who were frail or had probable dementia, highlighting the potential value of these geriatric conditions in identifying those at increased risk.

## Notes

Supplement 1. **eFigure 1.** Assembly of Analytic Sample **eTable 1.** Rates of Hospital Readmissions After Major Surgery According to Demographic, Surgical and Geriatric Subgroups from 2011 to 2018 **eTable 2.** Characteristics of Hospital Readmissions After Major Surgery **eFigure 2.** Top 10 Principal Diagnoses for Hospital Readmissions After Major Surgery

Supplement 2.
Data Sharing Statement
