# Supplementary material for: National Estimates of Short- and Longer-Term Hospital Readmissions After Major Surgery Among Community-Living Older Adults
Source: JAMA Netw Open. 2024 Feb 28;7(2):e240028. doi: 10.1001/jamanetworkopen.2024.0028 (PMC10902728; doi:10.1001/jamanetworkopen.2024.0028)
Supplement: Supplement 1. — eFigure 1. Assembly of Analytic Sample eTable 1. Rates of Hospital Readmissions After Major Surgery According to Demographic, Surgical and Geriatric Subgroups from 2011 to 2018 eTable 2. Characteristics of Hospital Readmissions After Major Surgery eFigure 2. Top 10 Principal Diagnoses for Hospital Readmissions After Major Surgery [file jamanetwopen-e240028-s001.pdf]

Supplementary Online Content

Wang Y, Leo-Summers L, Vander Wyk B, Davis-Plourde K, Gill TM, Becher RD. National estimates of hospital readmissions after major surgery among community-living older adults. *JAMA Netw Open*. 2024;7(2):e240028. doi:10.1001/jamanetworkopen.2024.0028

eFigure 1. Assembly of Analytic Sample

eTable 1. Rates of Hospital Readmissions After Major Surgery According to Demographic, Surgical and Geriatric Subgroups from 2011 to 2018

eTable 2. Characteristics of Hospital Readmissions After Major Surgery

eFigure 2. Top 10 Principal Diagnoses for Hospital Readmissions After Major Surgery

This supplementary material has been provided by the authors to give readers additional information about their work.

© 2024 Wang Y et al. *JAMA Network Open.*

eFigure 1. Assembly of Analytic Sample

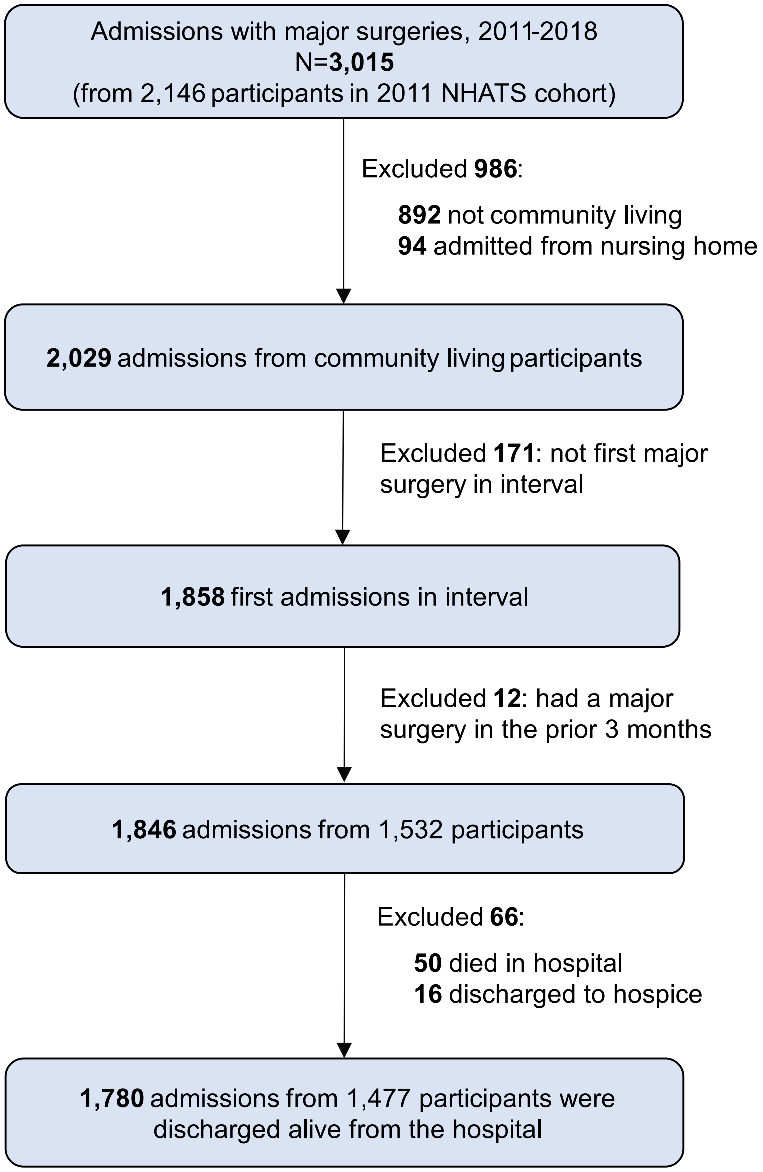

© 2024 Wang Y et al. *JAMA Network Open.*

eTable 1. Rates of Hospital Readmissions After Major Surgery According to Demographic, Surgical and Geriatric Subgroups from 2011 to 2018.

Subgroups	30 Days (n=9,556,171)		180 Days (n=9,458,666)	
	Number of Readmissions	Rates (95% CI), %	Number of Readmissions	Rates (95% CI), %
Total	1,105,935	11.6 (9.8-13.6)	2,608,373	27.6 (24.7-30.7)
Age group, year				
65-69	94,287	10.3 (5.4-18.6)	196,577	21.4 (12.7-33.8)
70-74	287,958	10.5 (7.3-14.9)	670,086	24.8 (19.9-30.4)
75-79	347,816	13.5 (10.2-17.5)	762,158	29.8 (25.2-34.8)
80-84	205,155	11.7 (8.4-16.1)	512,672	29.5 (23.7-35.9)
85-89	115,053	10.7 (7.7-14.7)	288,244	27.2 (20.7-34.8)
≥90	55,666	11.4 (6.8-18.5)	178,636	36.8 (28.3-46.3)
Sex				
Male	555,967	12.7 (10.3-15.5)	1,258,953	29.2 (25.4-33.2)
Female	549,968	10.6 (8.5-13.2)	1,349,420	26.2 (22.8-30.0)
Race and ethnicity				
Non-Hispanic White	898,542	11.1 (9.2-13.4)	2,203,612	27.6 (24.4-31.1)
Non-Hispanic Black	97,713	14.3 (10.1-19.8)	217,074	32.1 (25.8-39.2)
Hispanic	46,330	11.6 (5.0-24.5)	77,546	19.4 (10.9-32.0)
Other	63,350	15.8 (7.9-29.0)	110,141	27.4 (15.8-43.3)
Medicare type				
Fee-For-Service	721,680	10.9 (8.7-13.6)	1,806,549	27.6 (24.1-31.4)
Medicare Advantage	384,254	13.1 (10.5-16.3)	801,824	27.5 (23.1-32.4)
Surgical				
Elective	562,038	9.9 (8.0-12.2)	1,432,692	25.4 (22.0-29.0)
Nonelective	543,897	14.1 (11.4-17.2)	1,175,681	30.9 (27.3-34.7)
Type of surgery				
Musculoskeletal	342,079	8.4 (6.4-11.0)	860,028	21.4 (18.0-25.2)
Abdominal	245,083	14.4 (10.4-19.5)	523,046	30.8 (24.7-37.7)
Vascular	170,003	17.6 (12.1-25.0)	431,173	45.8 (37.7-54.1)
Cardiothoracic	136,837	13.7 (8.2-22.2)	319,138	32.1 (24.3-41.2)
Neurologic	66,938	7.2 (3.5-14.3)	216,363	23.8 (16.6-32.8)
Other	144,994	16.0 (11.5-21.8)	258,625	28.7 (22.3-36.1)
Frailty phenotype				
Nonfrail	215,436	7.7 (5.2-11.3)	526,790	18.9 (14.8-23.8)
Prefrail	652,185	13.5 (11.4-16.0)	1,382,525	28.9 (25.5-32.6)
Frail	238,313	12.3 (8.6-17.3)	699,058	36.9 (30.8-43.5)
Dementia status				
No dementia	886,790	11.2 (9.3-13.3)	2,056,033	26.1 (23.2-29.3)
Possible dementia	104,174	12.3 (8.0-18.4)	255,001	30.7 (24.0-38.4)
Probable dementia	114,971	14.8 (10.4-20.6)	297,339	39.0 (30.7-48.1)

Abbreviations: n, number; CI, confidence interval.

All values are survey-weighted.

© 2024 Wang Y et al. *JAMA Network Open.*

eTable 2. Characteristics of Hospital Readmissions After Major Surgery

Characteristic	30 Days (n=1,105,935) Number of Readmissions	30 Days (n=1,105,935) Rates (95% CI), %	180 Days (n=2,608,373) Number of Readmissions	180 Days (n=2,608,373) Rates (95% CI), %
Unplanned	802,372	72.6 (66.0-78.2)	1,701,458	65.2 (59.9-70.2)
For major surgery	245,501	22.2 (16.5-29.2)	808,895	31.0 (26.6-35.8)
Length of stay, days				
Mean (SD)	5.4 (5.0)		5.4 (5.5)	
Median (IQR)	4 (2, 7)		4 (2, 7)	

Abbreviations: n, number; CI, confidence interval; SD, standard deviation; IQR, interquartile range.
All values are survey-weighted, except for length of stay.

© 2024 Wang Y et al. *JAMA Network Open.*

eFigure 2. Top 10 Principal Diagnoses for Hospital Readmission after Major Surgery

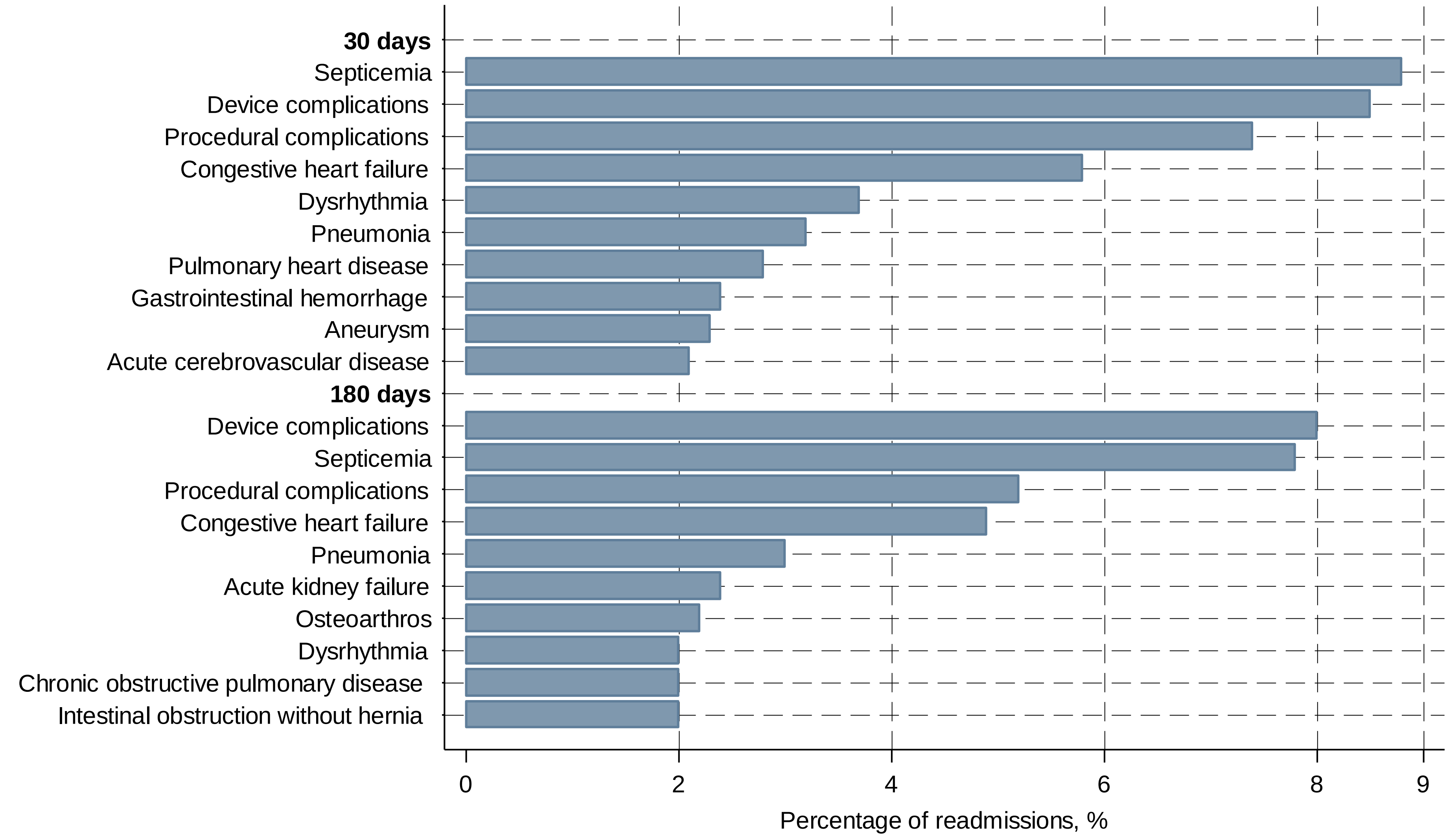

© 2024 Wang Y et al. *JAMA Network Open.*
