# Supplementary material for: National Estimates of Short- and Longer-Term Hospital Readmissions After Major Surgery Among Community-Living Older Adults
Source: JAMA Netw Open. 2024 Feb 28;7(2):e240028. doi: 10.1001/jamanetworkopen.2024.0028 (PMC10902728; doi:10.1001/jamanetworkopen.2024.0028)
Supplement: Supplement 2. — Data Sharing Statement [file jamanetwopen-e240028-s002.pdf]

# Data Sharing Statement

Wang. National Estimates of Short- and Longer-Term Hospital Readmissions After Major Surgery Among Community-Dwelling Older Adults. *JAMA Netw Open*. Published February 28, 2024. doi:10.1001/jamanetworkopen.2024.0028

**Data**
**Data available:** No

**Additional Information**
**Explanation for why data not available:** Because of DUA restrictions, we cannot provide external investigators with access to analysis-specific NHATS cohort study data that include sensitive/restricted files. Upon request, we will make the code that constructed files and/or ran analyses available to external investigators who wish to replicate our analyses. These investigators would need to apply to NHATS/CMS for access to the restricted CMS data.
